# Repertory of the Back

**Published:** 1899-05

**Authors:** E. H. Wilsey

**Affiliations:** Parkersburg, W. Va.


### REPERTORY OF THE BACK.

E. H. Wilsey, M. D., Parkersburg, W. Va.

Continued from April No., p. 189.

### Lumbar Region.

**Rheumatic** pains contracted by removing flannels too soon, Tereb.

— drawing and aching from loins to coccyx, *Carbo-veg*.

— pains of loins and hips extending to left shoulder and chest, *Colch*.

**Rheumatism** of back, lumbago, *Nux-v*.

— of lumbar muscles, *Phyto*.

— of lumbar muscles extends to or goes to the toes, Colch.

— of lumbar muscles, worse from every motion, *Bryon*.

— of lumbar muscles after getting wet while warm, stitching, drawing, *Dulc*.

**Rising**, pain on rising from a seat and beginning to walk, better by walking, Tabac.

— tearing in lumbar region rising, so that he became erect only gradually, Iris-v.

— lumbago all night, goes off on rising, *Ferr*.

— painful stiffness in loins when rising from a seat, *Led*.

**Rubbed**, lumbago and sciatica; the vertebræ feel as if rubbed against each other, *Ant-t*.

**Sciatica** and lumbago; the vertebræ feel as if rubbed together, *Ant-t*.

**Scream**, stitches in the lumbar vertebrae compelling him to scream, Phos.

— violent pressive pain in lumbar region, worse when at rest, so that she has to walk about to get better, but by coughing it is so much worse that she must scream, Nitrum.

**Sensation** as if a bladder filled with air in left supra-iliac region, *Bry*.

— of a heavy weight in the lumbar and dorsal region, worse by

LUMBAR REGION.

lying on left side with increase of temperature and sensibility of parts affected, *Coloc.*

**Sensation** of cramp in left lumbar region, Bell.

**Sensitive** to touch in lumbar region, Med.

— slightly sensitive to pressure in lower lumbar region, Arg-n.

**Sensitiveness** of lumbar vertebræ, *Agari.*

— of lumbar muscles to touch, Bry.

**Separate,** pain as though lower lumbar vertebræ would separate, when bending forward, *Chelid.*

**Severe,** aching in lumbar and sacral region, Ptelia.

— pain in lumbar and hypogastric region extending down legs, *Ham.*

— pain in lumbo-sacral region, Amm-m.

— continuous aching in lumbar region, Lyco-v.

— pain in lumbar region with fever, Tereb.

— pain in lumbar and sacral region mostly during latter part of night of long standing, *Carbol-ac.*

— pain in back and loins, *Caulo.*

**Sewing,** pain in lumbar region all day while sewing, Iris-v.

**Sexual** excitement constant with pain in lumbar region, severe erections, numbness and tingling in feet and legs, Onos.

— pain across loins as if broken, with loss of sexual power, *Coccul.*

**Sharp** pains toward sacrum from lumbar region, Sul.

— stitching pains, from loins and nates, pulse weak and soft, awakens him at 3 A. M., he must get up and walk about, *Kali-c.*

— pains in loins, Kalmia.

— pain first in lumbar region, then in left leg extending up thigh, then in right; feels weak as if over-fatigued, Lyco-vir.

— merely on inspiration a sharp piercing pain in right loin, Aur-fol.

— lancinating pain in lumbar region, Senecio.

— stitches in left loin by the last false rib, Caustic.

— pain in lumbar region extending to testes, Diosc.

LUMBAR REGION.

**Sharp,** sudden sharp pain in right lumbar region in the morning during forcible expiration when standing, Coca.

**Shock-like,** in left loin a shock-like motion downward as from a dropping fluid, Zingiber.

— a wave-like shock above left loin, Stann.

**Shooting** and sudden tearing in lumbar region, *Colch.*

— frequent in left lumbar region, Mag-m.

— or aching pain in lumbar region, Jug-cin.

— down legs and aching in loins, *Sil.*

— forward in left loin while sitting, Still.

— pressive shooting pain on the external right side of the lumbar vertebræ, worse in morning, Mez.

**Shudders,** burning itching, like a flea bite in region of left loin so that he shudders, Alum.

**Sitting,** bruised feeling in region of loins especially while lying and sitting, Agari.

— shooting forward in left loin while sitting, Still.

— tired feeling in right lumbar region in the evening while sitting in church, Ferr-phos.

— violent pains in the loins when she rises after sitting, Carbo-an.

— bruised pain in lumbar region, worse by sitting bent; stitches, *Kali-iod.*

— bruised sensation when sitting and standing, Sul-ac.

— pain in lumbar region, worse by sitting and lying, Tabacum.

— pain in loins when sitting as before menses, Carbo-an.

— burning and tired aching in lumbo-sacral region on sitting. *Helon.*

— pain when sitting as if back had been bent too long, and as if one had been lying on it too long, Rhod.

— drawing in lumbar region (right), worse walking, better sitting, Æthusa.

— obtuse stitch outward, kind of forcing out, in both loins at every inspiration, while sitting bent forward, Dulc.

### LUMBAR REGION.

**Sitting,** aching around loins from long sitting and when rising at night, Pallad.

— digging stitches in left loin, disappearing when walking, but recurring when sitting, Dulc.

**Smarting** and burning violently in lumbar region, and symptoms of slow hectic fever, *Phos-ac.*

— in lumbar region, Lac-c.

**Sneeze,** inability to sneeze or cough on account of pain in lumbar region, Nux-v.

**Sore** pain extending around abdomen from loins externally, Baryta-c.

— pain in lumbar region when bending over to either side, Cornus.

— and tense on touch in lumbar region, Nux-v.

— lame feeling in lumbar region, Onos.

**Soreness** in lumbar region, *Agari.*, *Caps.*, *Berb.*, *Baryta-c.*, Cimex., Brom., Nux., Æsc., Nat-c., Ptel., Lyss., Colch., Uran-n., Sul-ac., Eupat-per., Diosc., Conval., Cornus., Onos., Rhus-t.

— lumbar pain of three weeks standing after a strain; great pain and soreness, worse getting warm in bed and on beginning to move, Rhus.

— of lumbar region, in A. M., Diosc., Convall.

— on movement or like a drawing in lumbar region, Sul-ac.

— internally in lumbar region extending toward the abdomen, Nat-c.

— deep-seated pains in loins with soreness from motion, Eupat-per.

— lumbar pain, aching distress, soreness on waking or wakens him at 4 A. M., Ptelia.

— of last dorsal and first lumbar vertebræ, Bell.

— painful in lumbar region followed by frequent diarrhœic stools, *Baryta-c.*

**Sparks,** electric sparks like extending into ribs from above ilium, Caustic.

LUMBAR REGION.

**Spasmodic** lumbar pain, Copaiva.
— lumbar pain does not allow him to arise, *Sil.*
— lumbar pain by motion, cold and contact during menses, *Coccul.*
**Sprained** pain in lumbar region, Rhod.
— violent pain as if sprained in the lumbar region, and sacral; unendurable on adduction of left thigh compelling him to limp, Arg-met.
— pain in lumbar region after heavy lifting and taking cold at same time, *Sul.*
— pain in lumbar region in afternoon and evening in bed, Sepia.
— after lifting a heavy weight during pregnancy, Sec.
— as after over-lifting, worse while at rest, at night, and when rising from a seat, *Staph.*
**Squeezed,** pain as if squeezed in lumbar region, Caustic.
**Squeezing,** dull stitches from both loins with a sensation of a squeezing from within outward at every inspiration, after walking, Dulc.
**Standing**, pain with drawing through lumbar vertebræ on standing, Cann-i.
— sudden sharp pain in right lumbar region in morning during forcible expiration when standing, Coca.
— pain in loins like toothache when she stands, which makes her feel sick and good-for-nothing, *Colch.*
— tensive drawing and pinching in loins and above hips at noon when walking and standing, Ver-a.
— a paralytic sensation as from a weakened back in loins; worse by walking and standing, Agari.
— drawing through lumbar vertebræ when standing, Con.
— and walking better, weakness in lumbar region, Diosc.
— lumbago, crampy when standing in the streets without overcoat; better by external support and adjusting position, Chin-s.
**Stand,** weakness in lumbar region and lower limbs so he could scarcely stand alone, Sul-ac.

LUMBAR REGION.

**Sticking** in loins at noon, Hyperi.
— griping by false rib, Stann.
— in loins midway between hypochrondrium and iliac crests in evening when playing on piano, Stram.
— pain in lumbar vertebræ with every expiration, Sul.
— in lumbar region, extending to knee in morning, Psorin.
— in lumbar region, *Berb.*
— in lumbar region on deep breathing, Phelland., *Merc-s.*
— in left lumbar region; better during inspiration, Asaf.
— in lumbar region, extending to left lung, Sepia.
— to small of back, liver, and thence to urethra, Lach.
**Stiff** pain in loins after sitting, Cham.
— lumbago, back very stiff every morning, *Phyto.*
— muscles tender on pressure and stiff, especially on first moving after repose, *Cact.*
— pressure above the loins, with sensation in the legs as if they were stiff and bandaged, Nat-m.
— and lame feeling in back and lumbar region, Cup.
**Stiffness** in lumbar region, Amm-m., Onos., *Ledum*, Sul., Syph., *Carbo-an.*, *Cact.*, Cup., *Helon.*, Bry., *Hydrast.*, Anac., Apis., *Benz-ac.*, Berb., Diosc., Petrol., *Sepia*, Uran-n., *Kali-c.*, *Lyco.*, Lauroc., *Manc.*, Baryta-c., *Lach.*, Cham., *Rhus*, *Sil.*, *Phyto.*
— of lumbar region, with amenorrhœa, *Kali-c.*
— of lumbar region at night, *Lyco.*
— painful in lumbar region, *Mancin.*, Lauroc.
— painful on motion, *Bry.*
— painful into thighs, *Lach.*
— in lumbar region; must use arm to rise from seat, *Hydrast.*
— when rising from seat, *Sil.*, *Led.*
— a heavy, dragging, dull feeling in lumbar region, with stiffness and want of elasticity, Syph.
— tearing and tenderness in joints and muscles of lumbar region, prevents motion and stooping most when standing or sitting, better when lying, Bry.

LUMBAR REGION.

**Stiffness** in lumbo-sacral joints, Caustic.
— of loins, Carbo-an.
— drawing and pressing in lumbar region, while bending over for a short time causing great difficulty when assuming an erect position, *Hydrast.*
— pains in lumbo-sacral region, with lameness and stiffness, *Helon.*
— aching across lumbar region, with stiffness, Onos.
— painful in loins when rising from a seat, *Led.*
— in lumbo-sacral region, which made it difficult to lean forward and forced him to stretch, Sul.
**Stinging** in lumbar region, Lyss.
— under left rib in lumbar region, Sars.
**Stitch,** dull, outward in left loin, close above the hip at every breath, Dulc.
— dull in right lumbar muscles, Spong.
— tensive in right loin, felt only during inspiration, most violent when lying on back, Coloc.
— in lumbar region when walking, *Ran-scl.*
**Stitches** in lumbar region, *Acon.*, Agari., Arund., *Berb.*, *Bry.*, *Cepa.*, *Colch.*, Dig., *Hyper.*, Ign., Iod., *Kali-iod.*, *Magn-m.*, Niccol., Plumb., *Puls.*, Spong., *Sul.*, Zing., Con., Amm., Amm-c., *Cocc-c.*, Dulc., Bov., Carbo-a., Lauroc., Canth., Cajup., Cann-s., Nat-m., Elater., Enony., Caust., Sep., *Ran-b.*, *Ran-scl.*
— in left abdomen toward lumbar region, *Calc-c.*
— with cough *Amm-c.*, *Bry.* (in lumbar region).
— when coughing breathing deeply or walking, Arn.
— during cough, better by pressure on lumbar region, Bor.
— dull in right lumbar region, Spong.
— in lumbar region in gastritis, *Coccul.*
— in right hypochondrium towards lumbar region, *Calc.*
— during inspiration in lumbar region, Amm.
— into limbs from lumbar region, Stann.
— worse from motion, sciatica, *Calc.*

### LUMBAR REGION.

**Stitches** in lumbar region when moving and better by pressure, *Dulc.*

— in lumbar region from ovaries, *Coloc.*

— in lumbar region, worse in turning body, Bov.

— above loins on deep breathing, extending into thighs with every breath, Carbo-a.

— cutting worse when sitting afternoon, extending to beneath shoulders, where it becomes stitching, Canth.

— alternating above right hip and lumbar region, Carbo-a.

— through lumbar region when stooping, Cajup.

— slow intermittent stitches in left side beneath lowest ribs, Cann-s.

— severe in lumbar region when breathing deeply, Nat-m.

— dull in both loins, with sensation as of squeezing from within outward at every inspiration when sitting bent, Dulc.

— fugitive stitches in lumbar region more on right side, Elater.

— in first lumbar vertebræ near left side, of spine, about middle of body, Euonymus.

— in back and lumbar region, Caust.

— sudden stitches in lumbar region when lifting, he had to walk bent forward, and had stitches if his foot struck against anything, Sepia.

— and tensive drawing pains in back much increased by moving arms upward, Con.

— in left lumbar region, worse by expiration, Amm.

— sudden in right loin, Amm-c.

— in lumbar vertebræ compelling him to scream, Phos.

— fleeting stitches in loins, *Cocc-c.*

— sharp in left loin by the last false rib, Caustic.

— dull in right loin only when not breathing, Clem.

— obtuse stitches outward, a kind of forcing out in both loins at every inspiration while sitting bent forward, Dulc.

— dragging stitches in left loin, disappearing when walking, but recurring when sitting, Dulc.

— in bones of lumbar region on expiration, Sul.

### LUMBAR REGION.

**Stitches** in right lumbar when walking, with a slight burning sensation, *Ran-b.*

— in right loin, Lauroc.

— through pelvis to external genitals, *Kreos.*

— from left lumbar region into penis, Dros.

— pulsating stitches in lumbar region, Sul.

— during rest, ceasing on walking, Staph.

— in right lumbar with nausea, *Nat-s.*

— in right lumbar with tension across abdomen, worse on motion, must sit forward with elbows on knees, face in palms, when walking, stooped forward with hands on knees (hepatitis), *Kali-c.*

— when sitting, in lumbar region, Amb., Asar., Kali-iod., *Nat-c.*, Lyco.

— in lumbar region when sneezing, Arundo.

— in lumbar region better after stool, Ind.

— when rising from stooping, *Lyco.*

— sudden in lumbar region, Cinnab.

— in lumbar region tearing, *Magn-m.*

— tensive in right lumbar region, felt only during inspiration, worse when lying on back, Coloc.

— with drawing through vertebræ while standing, *Con.*

**Stitching,** sharp pains from loins and nates, pulse soft and weak, awakens him at 3 A. M., he must get up and walk about, *Kali-c.*

— pain in small of back so severe upon motion that he gets upon arms and knees to obtain relief, pain unendurable in any other position, *Colocy.*

**Stool,** constriction in lumbar region, worse after stool, Tabac.

— drawing pain in lumbar region after stool, Mag-m.

— pain in lumbar and sacral region, worse during stool and worse still afterward, *Podo.*

— forcing in lumbar region as if to stool, jerking in region of hips, Ratanhia.

— violent cutting in lumbar region or vertebræ, worse by stool, Rheum.

### LUMBAR REGION.

**Stool**, pains in lumbar region before stool, *Puls.*
— pain in lumbar region during soft stool, Niccol.
— pain in lumbar region if he does not have a stool, *Cepa.*
— pressing in lumbar region after stool, *Carbo-v.*
— pain in lumbar region after stool, must walk for relief, *Lyss.*
— pain in lumbar region better after stool, Ox-ac.
— drawing pains in loins after stool, Mag-m.
**Stooping,** pain in loins preventing stooping, Bufo.
— pain in loins on walking and stooping, Hyos.
— stitches through lumbar region when stooping, Cajup.
— pain in lumbar region worse by stooping, Rhod.
— pain in lumbar region worse by protracted stooping, so that walking is impeded, better by rest when sitting or lying, reappearing as stitches on slightest turning of body, better afternoon, Sarsa.
— pain, violent, tensive as if everything was too short when stooping, Sul.
— pain, as after stooping a long time, *Dulc.*
— heavy pain in lumbar region, worse by stooping, Diosc.
**Straighten,** must walk about sometimes before he can straighten up, *Hydras.*
**Strain,** lumbago from strains: pains after rest after moving a little, and from warmth (when Rhus does no good), *Calc-fl.*
— lumbar pain of three weeks' duration after a strain, Rhus-t.
**Strained,** pain as if strained in lumbar region, preventing him from straightening himself or stooping during the pain in the loins; the urine is scanty, of a yellowish ochre color, thick with a yellowish sediment, Bufo.
— intense pain in left lumbar region above hips as if he had strained parts by standing, Valer.
**Sweat**, crampy pain in right loin in evening causing sweat, Iris-v.
— cold in lumbar region, Plant.
— in lumbar region, *Sil.*, Asaf.
**Swelling**, thick swelling of lumbar muscles, very painful when the body is moving, *Lyco.*

### LUMBAR REGION.

**Swelling** of lumbar region, *Aur-Mur.*
— of lumbar muscles extending to legs, Berb.
— of lumbar region in acute Bright's disease, *Apis.*
**Tearing**, in lumbar region, *Æsc-h.*, Agari., Aur.-fol., Berb., Brom., Bry., Cinnab., Cup-met., Chel., *Caustic.*, *Colch.*, *Ham.*, *Mag-m.*, *Kali-c.*, Polyg., Rhus.
— across lumbar region after heat, Ham.
— worse on one side in lumbar region, Berb.
— pain now in right side now in left side of lumbar vertebræ, while walking, Agari.
— with pressure in lumbar region, Carboneum.
— pains commencing along lumbar vertebræ, running forward around left lumbar region to linæ alba, *Caustic.*
— in lower lumbar vertebræ extending to iliac bones, Chel.
— in hips and lumbar region when walking, *Æsc-h.*
— and shooting suddenly in loins, *Colch.*
— sudden in lumbo-sacral region in afternoon, Sul.
— in lumbar muscles impeding respiration, *Kali-c.*
— drawing on bone in lumbar region, Rhod.
— in lumbar region on rising so that he can become erect only gradually, Iris-v.
— fine tearing, lancinating on right side beside the lumbar vertebræ, passing away every time he presses upon it, Aur-fol.
— in right lumbar and dorsal muscles, worse by moving those parts, Brom.
— pain in loins with arthritic tearing in lower limbs, Ver-alb.
— and tenderness in joints and muscles, lumbar region prevents motion and stooping, most when standing or sitting, better when lying, *Bry.*
**Tender**, pain in loins seems to rise in a spot which is tender to pressure about an inch to right of middle of spine, Chromic-a.
— muscles tender on pressure and stiff, especially on first moving after repose, *Cact.*

### LUMBAR REGION.

**Tension,** lumbar region so he could not breathe easily, Nit-ac.
— in lumbar region, *Zinc.*, *Nat-m.*, *Nux-v.*, *China.*, Berb., Aur-mur., Agar.
— painful in lumbar region, *Puls.*, Sul., Baryta-c., Amb., Benz-ac., Thuja.
— in lumbar region, worse going up stairs, Carb-s.
— in lumbar region, worse stooping, Clem.
**Tensive** pain in right loin; only perceived during inspiration and most severe while lying on the back, *Colocy.*
— pain in lumbar region, very violent, and in shoulders, Zinc.
— drawing in lumbar region, reaching sometimes to testicles, Psorin.
**Testicles,** pain in lumbar portion of spine with myalgic inflammation of testicles, Medor.
— pain in lumbar region at 5 P. M., extending to testicles, Abrot.
**Throbbing** pain in lumbar region, *Nux-v.*, *Sepia.*, *Coloc.*, *Sil.*, Nat-m., Thuja., Lac-can., Alum.
— pain in lumbar region, with eructations during shaking chill, *Nux-v.*
— dull pain in left lumbar region, worse by lying down; was compelled to walk about room with walking-stick pressed across back to obtain relief, Vib-op.
— in lumbo-sacral region, *Sil.*
— painful in lumbar reigion, Actea-rac., Nat-m.
— in lumbar region during stool, Alum.
**Thrust,** dull with sensation of external coldness against him, Stann.
**Tired** aching over crest of ilium and down into pelvis; worse in right side, with general languor and desire to lie down; later the tired aching, worse morning and A. M., Spig.
— aching in lumbar region on awaking, Pic-ac.
— pain, pressing, drawing in lumbar region, *Sepia.*, *Mur-ac.*
— and weak and aching across lumbar region, *Helon.*
— sensation in lumbar region, Carbol-ac., *Pallad.*

### LUMBAR REGION.

**Tired** feeling in lumbar region (right) while sitting in church, Fer-phos.
— in lumbar region, Senecio.
— feeling when walking, Camph.
— painful feeling in lumbar region, *Puls.*
— painful feeling in lumbar region with uterine disease, Murex.
— feeling; is obliged to sit down, Cimex.
— feeling in lumbar region, *Helon.*, Sabin., *Æsc-hip.*, Arg-nit., Chen-v., Cimex, *Coloc.*, Mancin., *Cina.*, *Pallad.*
**Tiresome** feeling as if bruised, *Sul.*
**Touch**, sore and tense on touch in lumbar region, Nux-v.
— lumbar vertebræ sensitive to touch, Med.
— spot on spine sore as if ulcerated very sensitive to touch, *Colchi.*
— sensitiveness of muscles of lumbar region to touch, Bry.
**Trembling** in lumbar region, Benz-ac., Berb.
**Turning** or bending, aggravates pain in left lumbar vertebræ, Ginseng.
— stitching in lumbar region by turning body, Bov.
**Twitching** in lumbar region at 2 P. M., Thuja.
— in lumbar region, *Coloc*, Agari, Ratanhia.
— visible of lumbar muscles, Crotal.
**Uneasy** feeling in lumbar region with a sensation as if a drop of urine were in penis, Cedron.
**Uneasiness**, indescribable in lumbar vertebræ, *Sabina.*
— in lumbar region in evening, Petrol.
**Unendurable** pain in loins and hip-joint at night if he lies upon opposite side, Cham.
**Ulcerating** pains in lumbar vertebræ as if flesh were being torn off, Kreos.
**Ulcerative** pains in lumbar region, Cann-s.
— pain in lumbar region at night so can only lie on right side, better in morning after getting up, Nat-s.
**Urging** in lumbar region, *Calc.*
**Urine**, pain in lumbar region on retaining urine, *Nat-s.*

LUMBAR REGION.

**Urinary,** violent writhing in region of loins and urinary passages, *Colch.*

**Violent** pain in loins when she rises after sitting awhile, Carbo-an.

— pain in loins and small of back, *Tabacum.*

— pain in first and second lumbar vertebræ when turning, with formication in feet, Agari.

— pain in lumbar and sacral region, slight effort to move causes retching and cold, clammy sweat, *Ant-t.*

— pain as if sprained in lumbo-sacral region, compelling him to limp, Arg-met.

**Vomiting,** with pain in loins, Ars-h.

— loins affected after vomiting, Therid.

**Waking,** a drawing pain waking him, worse on turning, *Bry.*

— dragging when waking in morning in lumbar region, also till P. M., Myrica.

**Walking,** pain in loins with giving away while walking, Bufo.

— pain in loins, better by walking, rest or pressure, *Phos-ac.*

— pain in loins on walking and stooping, Hyos.

— dull lumbar backache, worse by walking, *Bapt.*

— pain as from dislocation in loins and os ischia when sitting, and when turning the body in walking, Hepar.

— burning in a small spot on left side of lumbar vertebræ and same time in lower part of left scalpula, worse from rising from a seat, better when walking, Baryta-c.

— stitches in right loin when walking, with a slight burning sensation, *Ran-b.*

— stitch in lumbar region when walking, *Ran-scl.*

— burning in lumbar region, extending through thigh on walking, *Nux.*

— tearing pain, now on right side, now on left side of lumbar vertebræ while walking, *Agar.*

— with pressive drawing pain in small of back and loins only while resting (sitting, standing, or lying down), in daytime, disappearing on walking, Amm-c.

LUMBAR REGION.

**Walking,** digging stitches in left loins, disappearing when walking; but recurring when sitting, Dulc.
— pain on rising from a seat and on beginning to walk, better by walking, Tabac.
— tearing in hips and lumbar region while walking, *Æscul.*
— weakness, better by standing, Diosc.
— pain in lumbar region, worse by protracted stooping, so that walking is impeded, relieved by rest when sitting or lying; reappearing as stitches on slightest turning of body, relieved P. M., Sarsap.
— chronic lumbago of several years' standing, with difficult walking, *Oleum-j.*
— drawing pain in loins when walking, standing and lying, and feeling as if broken, *Carbo-an.*
**Walk**, sudden stitches in lumbar region when lifting; he had to walk bent forward, and had stitches if his feet struck against anything, Sep.
— dull aching in lumbar and sacral region; cannot walk, muscles will not obey, *Gels.*
— must walk about some time before being able to straighten up, *Hydrast.*
— pain as from a knife through loins, cannot walk, *Kali-c.*
— cutting in lumbar region, so could not walk alone, Psorin.
**Warmth,** lumbago from strains, pains worse after rest, better after moving a little and from warmth, *Calc-fl.*
**Washing,** lumbar backache after washing, *Podo.*
**Water,** sensation of water dropping out of a bottle in lumbar region, between posterior and superior spine of the ilium and vertebræ, Medor.
**Weakness** of lumbar region, *Sepia.*, *Ars.*, *Æscul-h.*, *Agari.*, Arum-d., Benz-ac., *Calc.*, Chen-v., Cimic., Chromic-ac., *Eupat-per.*, *Kali-b.*, *Kali-c.*, *Nux-m.*, Petrol., *Arg-n.*, *Ox-ac.*, *Ruta.*, *Selen.*, Thuja, Colch., *Zinc.*
— in lumbar region, Psorin.
— painful in loins after running too fast, Raphanus.

LUMBAR REGION.

**Weakness** and pain in lumbar region, *Amyl-n.*
— and dull aching in lumbar region, with hemorrhoids, *Æsc-hip.*
— in lumbar region, with tension in head, Zinc.
— from lumbar region to legs, *Ox-ac.*
— of lumbar region follows seminal emissions, with lascivious dreams, waking him, Selen.
— when walking, from uterine trouble, *Sepia.*
— and heavy pain in lumbar region, Lil-tig.
— in loins after exercise, Plant.
— in lumbar region with prolapsus recti, *Ruta.*
— in lumbar region, Sabina.
— in lumbar region, better by standing and walking, Diosc.
— and heavy dragging pain in lumbar region, Eryngium.
— and trembling pain in lumbar region, Cimic.
— in lumbar region and lower limbs, so he could hardly stand alone, Sul-ac.
— a paralytic sensation as from weakness in back and loins, worse by walking and standing, Agari.
— of lumbar region after riding, Berb.
— of lumbar region with waning of sexual desire, from over-indulgence, *Sul.*
— on walking, in lumbar region, *Graphites.*
— in lumbar region with discharge of acrid mucous stools during urination, *Puls.*
—at junction of lumbo-sacral vertebræ, *Phos.*
—early on rising like paralysis, *Nat-m.*
— in lumbar region at 6 P. M., *Sepia.*
— in lumbar region with irritable bladder, Sepia.
**Weight** and pain in lumbar region extending around body, *Cimicif.*
— sensation of heavy weight in lumbo-dorsal region, *Coloc.*
— sense of great weight across loins, *Arn.*
— and pain in lumbar region, Kali-chl.
**Withered** pain in lumbar region, sensation of worse at night, Podo.
**Writhing** violently in region of loins, *Colchi.*

